# A Blockchain-Assisted Intelligent Transportation System Promoting Data Services with Privacy Protection

**DOI:** 10.3390/s20092483

**Published:** 2020-04-27

**Authors:** Yuhong Li, Kun Ouyang, Nanxuan Li, Rahim Rahmani, Haojun Yang, Yiwei Pei

**Affiliations:** 1 State Key Laboratory of Networking and Switching Technology, Beijing University of Posts and Telecommunications, Beijing 100876, China; oyk10@bupt.edu.cn (K.O.); 18401626082@163.com (H.Y.); peiyiwei@bupt.edu.cn (Y.P.); 2 Key Laboratory of Embedded System and Service Computing, Ministry of Education, Tongji University, Shanghai 201804, China; 3 Department of Science, University of Melbourne, Melbourne 3010, Australia; nanxuanl@student.unimelb.edu.au; 4 Department of Computer and Systems Sciences, Stockholm University, 16407 Stockholm, Sweden; rahim@dsv.su.se

**Keywords:** intelligent transportation system, vehicular networks, blockchain, security and privacy protection, data services

## Abstract

Being able to obtain various environmental and driving data from vehicles is becoming more and more important for current and future intelligent transportation systems (ITSs) to operate efficiently and economically. However, the limitations of privacy protection and security of the current ITSs are hindering users and vehicles from providing data. In this paper, we propose a new ITS architecture by using blockchain technology solving the privacy protection and security problems, and promoting users and vehicles to provide data to ITSs. The proposed architecture uses blockchain as a trust infrastructure to protect users’ privacy and provide trustworthy services to users. It is also compatible with the legacy ITS infrastructure and services. In addition, the hierarchical organization of chains enables the scalability of the system, and the use of smart contracts provides a flexible way for introducing new services in the ITS. The proposed architecture is demonstrated by a proof of concept implementation based on Ethereum. The test results show that the proposed architecture is feasible.

## 1. Introduction

Intelligent Transportation System (ITS) aim at facilitating urban mobility economically and ecologically by connecting vehicles with the network infrastructure and therefore using various information services. In general, five types of services are expected to be provided by ITS, namely: (i) intelligent transportation. For example, through intelligent routing planning and navigation, an optimistic route in terms of time and economy can be selected, avoiding traffic jams, etc. (ii) Safe driving assistance. With the aid of connected vehicles, early alarms about obstacles or vehicle breakdowns can be sent to the users, automated or autonomous vehicles. (iii) Driver or vehicle-related services, such as fines for breaking traffic rules, automatic maintenance of vehicles. (iv) Infotainment for people on board vehicles, such as streaming video, etc. (v) Providing environmental data, including traffic data obtained through crowd sourcing. 

With the development of big data technology and the increasing capability of vehicles’ on-board computers and sensors, collecting and providing data by vehicles has become very important for ITS to operate efficiently and economically, and for 3rd party service providers to provide both ITS and environmental monitoring-related services.

Nevertheless, currently collecting and providing data to ITS means also exposing the privacy of vehicles and their drivers, causing them face security risks too, since more and more data are related to users’ privacy, such as location, activities, model of cars and even traffic rules broken, etc. while no fundamental measures have been taken to protect users’ privacy. Some services provided by ITS, such as navigation and route planning may also greatly expose users’ privacy while bringing conveniences to users. Even the automated and autonomous vehicle-related decision making, controls and cooperation are dependent on the data obtained through networks, where security problems have been found [1].

The basic reason for that is the current ITSs are mainly based on the existing network infrastructure, where privacy and security protection have not been considered when they were designed. Moreover, the users and services of ITS are very heterogeneous. Human beings, vehicles and various services providers, etc. can all be involved in the ITS. If the security and privacy-related methods are complex, for instance, complicated authentications and encryptions are used, the services will not be widely accepted and used by users, especially data-intensive services like crowd sourcing. In general, the following problems exist in the current ITS architecture related with security and privacy protection:

- Over-dependency on centralized clouds. Current ITS architectures and services are based on centralized clouds where vehicles and users are identified, authorized and authenticated, and services are provided. In such architectures the cloud servers may become a bottleneck and a single point of failure that can disrupt the whole network, and a target for security attacks and the source of privacy leaks. Moreover, the centralized architecture and data storage are not scalable as ever larger numbers of vehicles are connected.
- No discrimination of data. In the current vehicular networks, the privacy-sensitive data have not been discriminated from the environmental monitoring data. Service-related data are processed and stored along with the data used for crowd sourcing. This is because the users’ and vehicles’ identities are closely correlated with the data they send to the networks through IP addresses and users’ private information stored by the service providers, etc. Especially, very often the environmental information and vehicles’ states data integrated with users’ identities are sent to the cloud without the owner’s permission; while using customized services means detailed private information must be provided to the service providers.
- High overhead for provisioning secure and privacy-protecting services. Although some secure services can be provided in the current network infrastructure and ITS, the complex key management, long and complicated authentication, authorization and access control procedure, and the high cost prevents people from using the secure services.
- Difficult to trace malicious behaviours. Modern vehicles are becoming more and more dependent on the software and control functions on board. An attack on the software or control functions of a vehicle (e.g., installing a malicious software online) may cause serious problems on the safety of the drivers and passengers. However, the malicious behaviours from both services providers or other users cannot be well traced and found in time.

Blockchain, as a distributed ledger technology, is a new distributed storage and computation pattern. Its inherent features of distributed trust, anonymity, data integrity and availability [2,3] provide a great potential for solving the problems in the current ITS. Thus, blockchain technology can benefit ITS in that the privacy, transparency and security can be dealt with by the ITS infrastructure itself. It can also lower the costs of the whole system operation. Based on this consideration, some work has begun trying to introduce blockchain into vehicular networks. For example, the goal of the cooperative project between Toyota and MIT [4] is to allow businesses and consumers to securely share driving and autonomous vehicle testing data, manage ride-share and car-share transactions, and store vehicle usage information that could be used in setting insurance rates. The authors in [5] proposed a blockchain-based anonymous reputation system (BARS) to establish a trust model for VANETs to prevent distribution of forged messages while preserving the identity privacy of vehicles. In [6] increased privacy and integrity as well as improved security that can be brought by blockchain to vehicular networks was discussed and solutions to the automotive security and privacy problems by using blockchain were suggested. Although the above work has tried to introduce blockchain into vehicular networks, only some specific functions are realized by using blockchain technology. No general method for providing secure services and privacy protection in vehicular networks has been given. 

In this paper, we propose a new ITS architecture integrating blockchain technology. In this architecture, hierarchical blockchains are constructed as overlays on top of the vehicular network infrastructure. No new network entities are introduced and therefore the backward compatibility of the legacy services in the ITS can be ensured. The anonymity and decentralization of the blockchain are used to protect privacy of users and reduce the single point failure of networks and services. The immutability of blockchain guarantees also the integrity of data and provide traces of malicious behaviours in the ITS. The smart contract of blockchain makes it easy and fast for deploying new services in ITS. Meanwhile, the efficient data processing and transmission of legacy vehicular networks are kept. Our goal is to stimulate vehicles and users to be involved in the ITS deeply by contributing and using data. The main contributions of the paper lie in the following aspects:

- We propose an architecture and mechanisms to integrate the blockchain technology into the current ITS. The proposed blockchain-assisted ITS (Ba-ITS) not only can provide privacy protection to users and an incentive mechanism which encourages users to contribute data to ITS, but it is also backward compatible with the legacy ITS.
- A hierarchical blockchain structure is suggested in the proposed Ba-ITS, enabling scalability for Ba-ITS. The interoperability among different levels of blockchains is considered.
- Methods for providing new services based on smart contracts are elaborated with examples, where legacy messages and blockchain-related messages are combined to provide services efficiently.
- We implemented a prototype of the proposed Ba-ITS based on Ethereum [7], and evaluated it by realizing two example services and simulations in ns3 [8].

The rest of the paper is organized as follows: We summarize the related work about ITS infrastructure and the usage of blockchain technology in ITS in Section 2. In Section 3, we elaborate the proposed Ba-ITS, concentrating on the hierarchical blockchain and how the services are provided in the Ba-ITS. Following this, we will explain our prototype implementation and give some performance evaluation results in Section 4. Finally, we will conclude our paper and describe our future work plans in Section 5.

## 2. Related Work

We discuss the related work in terms of data privacy and security in ITS infrastructure networks, the blockchain technology and its applications, as well as the usage of blockchain in ITS infrastructure networks.

### 2.1. Security and Privacy in ITS Infrastructure

Security and privacy protection have been identified as major requirements in vehicular networks, the basic infrastructure of ITS. Various protocols have been proposed to prevent vehicles and users from being identified from the exchanged messages. In [9], anonymity, confidentiality, conditional privacy, unlinkability, minimum disclosure and distributed identity resolution were discussed. The authors in [10] proposed an authentication scheme for VANET to verify the authenticity of the on-board computer (OBC) units of vehicles without revealing their real identities for V2V communications. Although an anonymous authentication is realized based on anonymous certificates, and the high computational cost can be reduced in the certificate revocation list (CRL) checking process and in the certificate and signature verification process, a central trust authority is needed and is responsible for maintaining the whole vehicular network. The authors in [11] proposed a conditional privacy preserving authentication scheme based on group signatures supporting batch verification to increase the efficiency of the authentication procedure. However, the verification delay and average data response delay still need to be improved. In [12], road side units (RSUs) are used to provide multiple anonymous keys for each vehicle in order to protect its communication from unauthorized users. However, big latency is introduced for completing the pseudonym generation. In [13], a group signature is generated for proving authentication to a group of vehicles, but when using this method, each vehicle needs to store the revocation list to avoid communication with revoked vehicles. Thus, the verification process grows linearly with the increase of the number of the revoked vehicles in the list.

In general, the current methods use an add-on authentication mechanism to prevent users from being easily identified from the exchanged messages and try to increase the efficiency in the computing procedures, since the underlying infrastructure does not provide any mechanisms for security and privacy protection. Compared with these methods, we have the same security and privacy requirement as identified in [1,5]. However, we try to solve the security and privacy problem intrinsically from the perspective of ITS architecture by using the blockchain technology. We introduce a blockchain overlay on top of the current ITS architecture to deal with the security and privacy protection. In this way, the information sent by users and vehicles is anonymous, and the data integrity can be guaranteed during transmission in networks. 

### 2.2. Blockchain and its Applications for Improving Security and Privacy

As a distributed ledger technology, blockchain is a hot research topic in recent years. Generally, the blockchain has the following inherent features [14]:

- Decentralized data storage and control: the exchanged data (blocks) are stored in different nodes across the blockchain network in the append-only way. No central authority dictates the rules.
- Data transparency and auditability: a full copy of each message exchange (transaction) executed in the blockchain is recorded in the blocks and is public to all the peers in the same chain. All transactions in the network can be traced.
- Decentralized consensus: only when a consensus on new transactions is achieved among the members in the blockchain can a new block be appended in the chain. This is different from the traditional databases, where the input data are assumed accurate until it is used.
- Anonymity and encryption: public/private key cryptography is used in blockchain. The source and destination of a transaction are identified by users’ public keys. In this sense, users’ anonymity can be guaranteed. In addition, the transactions are signed by the private keys of users and the content is hashed, this provides also a certain level of security.
- Autonomous program execution: a program (i.e., smart contract) can execute automatically with or without using the stored data in the network when predefined conditions are fulfilled. Smart contracts provide the network the ability of decision making without human being’s intervention.

Due to its inherent unique features, blockchain technology has been used in many areas, such as economy (e.g., microfinance, international trading, and digital rights) [15] and Internet of Things (IoT) [16]. In particular, blockchain has been used to provide security and privacy in IoT [17,18]. For example, in [17], smart homes constitute an overlay network together with service providers, cloud storages, and users’ smartphones or personal computers. Nodes in the overlay are grouped into clusters and each cluster has a cluster head. The cluster heads maintain a public blockchain in conjunction with two key lists. The requester key list maintains the users’ keys allowed to access data for the smart homes connected to this cluster; and the requestee key lists are the lists of keys of smart homes connected to this cluster allowed to be accessed. The IoT devices in a smart home are identified by device IDs. For each smart home, a smart home miner is used to process centrally the incoming and outgoing transactions to and from the smart home. Compared with this work, we share the same idea of having a blockchain overlay. However, in our architecture, each vehicle has its own public and private key pairs, and we do not rely on a central miner and cluster heads to maintain the keys. In addition, services are provided by using smart contracts, which can be traced and are more trusty.

In [18], IoTChain, a blockchain security architecture for IoT is proposed. The architecture is a combination of the Object Security Architecture for the Internet of Things (OSCAR) and the ACE authorization framework, aiming at providing an E2E solution with the secure authorized access to IoT resources. In the proposed architecture, the blockchain replaced the single ACE authorization server to handle authorization. The authorization servers, key servers, and clients act as blockchain nodes. Each client and resource owner is identified by at least one blockchain address. The blockchain handles the authorization requests through smart contracts. Compared with this work, we use the trustworthy nodes from the network infrastructure with high capacity as blockchain nodes. We use blockchain and smart contract to store and process information of anonymous users, and provide data services to vehicles and their users. Depending on the services, authorization can be realized implicitly through trusty blockchain nodes.

The H2020 GHOST project [19] aims to address different security and privacy concerns of IoT device installations in smart homes. In this context, the authors in [20] propose a method for signing a consent form through a distributed App (dApp) [21] connected to a set of smart contracts deployed in the GHOST blockchain network, so that certain functions of the network can be used by the users. In other words, the network gains trust from users in this way. Compared with this work, we use different smart contracts to deploy various services. Users can use dApp to obtain and display information about the services.

There are already several implementations of blockchain, such as Bitcoin [22], Ethereum [23], Hyperledger Fabric [24] and Corda [25]. Among them, Ethereum provides flexible interfaces and supports smart contracts, which make it easy to implement decentralized applications. The Ethereum and its built-in language Solidity have become the de facto standard. Our prototype is implemented using Ethereum 2.0.

### 2.3. ITS Based on Blockchain

Due to the unique features of blockchain, some work has been done trying to introduce blockchain into the vehicular networks, the basic communication infrastructure of ITS. 

The authors in [5] proposed a blockchain-based anonymous reputation system (BARS) to establish a trust model for vehicular ad-hoc networks (VANETs) to prevent distribution of forged messages while protecting the identity of vehicles. To protect the identity, a certificate authority (CA) operates the certificate issuance and public key revocation in order to eliminate the link between the public key and the identity of a vehicle. All the activities of CA are recorded in the extended blockchain transparently. In our architecture, we do not use a CA and certifications. The vehicles maintain the private key and public key for data transmission, such the legacy data transmission using the IP address is kept. 

In [6] the authors suggested a decentralized privacy-preserving and secure blockchain-based architecture for the smart vehicle ecosystem based on a lightweight scalable blockchain, which has low overhead. We share the same idea that each vehicle (owner) has finer control over the exchanged data. In other words, the vehicle owner can define what data can be shared to other users through the network, and what data are private and should not be exposed to other users. However, in our architecture, the private data are not stored in the in-vehicle storage but in the blockchain. In addition, in [6] each vehicle has its own overlay block manager (OBM). Mobility management mechanism (soft handover) needs to be used when a vehicle is far away from its OBM. In our architecture, we use the base stations (we call it a gateway) that a vehicle is currently connected as nodes on the blockchain to forward the transaction, which eliminates the handover procedure and reduces the latency for data transmission. 

The authors in [26] proposed an intelligent vehicular trust node (iv-tp) issued by the vehicle seller or an authorized dealer to establish the trust in the communication network. An authorized institution generates a unique ID and distributes it to a vehicle. By using blockchain, the complete history of the iv-tp node can be recorded, including the integrity value and criminal history. In [27] an application of charging through smart contracts by autonomous vehicles is proposed. In [28], a scenario of using blockchain for payment is proposed. During payment, information is exchanged through blockchain transactions, and finally automatic payment is achieved through a smart contract. In general, the above work implements specific functions by using blockchain technology. We aim at designing a new ITS architecture where various secure services with privacy protection can be provided flexibly and rapidly. 

Table 1 summarizes the major related work regarding the traditional ITS with security and privacy improvement and the ITS based on blockchain in terms of privacy, trust and security. 

Compared with the above works, we propose an ITS architecture assisted by blockchain, providing inherent security and privacy protection for data transmissions and for users. At the same time, new services can be introduced flexibly and rapidly. Furthermore, we use a hierarchical chain in the architecture, which makes the architecture scalable and suitable to vehicles with different capabilities. The interoperability between the neighbor layer chains are considered. 

## 3. Blockchain-Assisted ITS

As shown in Table 1, the major problems of current ITS based on blockchain are that they did not consider the scalability of the ITS architecture and the high computing and memory etc. requirement of the blockchain nodes. To deal with these challenges, we introduce an ITS architecture with blockchain overlay, where the blockchains are organized in three layers and different types of blockchain nodes can co-exist in order to provide the scalability and reduce the requirement on the capability of blockchain nodes.

### 3.1. ITS Architecture with Blockchain Overlay

**Figure 1:**
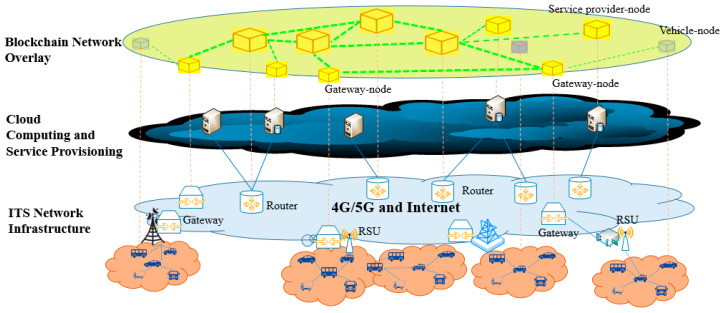
The ITS architecture with blockchain overlay.

Figure 1 illustrates the blockchain-assisted ITS (Ba-ITS) architecture, where blockchain network is an overlay consisting of the nodes in the ITS infrastructure and servers of ITS operators and other service providers. 

The architecture is composed of three layers—the ITS network infrastructure layer, the cloud computing and service provisioning layer and the blockchain overlay layer. The ITS network infrastructure layer consists of RSU (road side units), 4G or 5G cellular network base stations and Internet gateways and routers. Vehicles connecting to the ITS network belong also to this layer. Servers from ITS operators, for example those performing cloud computing and providing services, as well as servers from the third party service providers construct the service provisioning layer. The blockchain overlay layer is a virtual network consisting of some vehicles willing to join blockchain and use the security related services and some nodes from the ITS infrastructure and service provisioning layer needed for providing secure services with privacy protection. One of the important features of Ba-ITS is the backward compatibility with the legacy ITS system and services. Since the secure service can be realized by using overlays, the existing data communication and processing will not be affected, and the corresponding services can be kept.

#### 3.1.1. ITS Infrastructure and Service Provisioning Layer

The ITS infrastructure layer and service provisioning layer are accordant to the common, legacy three-tier structure - end, edge and cloud. According to the geo-graphical condition and the network environment, each vehicle can directly communicate with a 4G or 5G cellular network base station or with a RSU, which connects to directly via base stations to the Internet. Vehicles can also form into clusters according to certain clustering algorithms, and each cluster head connects to Internet via a RSU or a base station. Vehicles can also connect each other in an ad-hoc way, and one or several members connect to the Internet via a RSU and/or a 4G or 5G base station. Without loss of generality, in this paper we only discuss the case that every vehicle can connect to a RSU at any time, which connects to Internet via a gateway. To be convenient, we deem 4G and 5G base stations as gateways too. Hence, vehicles, RSUs, gateways construct a hierarchical network topology. The major network entities and their functions are as follows:

- Vehicular nodes: vehicles communicate with each other or to Internet by using IEEE 802.11p [29] or the corresponding dedicated short-range communications (DSRC) [30] and intelligent transportation system (ITS)-G5 [31]. In the context of ITS, vehicles have the functions of collecting, sending and receiving data. For instance, data about environment and traffic can be collected by sensors on board and uploaded to the cloud or edge nodes. Vehicles can also request data from cloud or edge nodes through RSU for route plan and autonomous driving etc. In addition, some vehicles may have intelligent control functions. Vehicles can configure what type of data uploaded to the network are privacy-sensitive, so that these types of data will be processed and stored in a trustworthy way.
- RSUs: RSUs communicate with vehicles using wireless interface and forward data between vehicles and Internet through 4G/5G base stations or Internet gateways in wireless or wired way. According to the hardware configuration, some RSUs can also perform certain computations on the data packets received from vehicles.
- Gateways: we call the routers at the edge of the core network forwarding and processing data from/to RSUs gateways. The 4G/5G base stations with certain computing and storage capability, or routers configured with certain computing and storage capability in the wired network connected with RSUs are all called gateways. The most important feature of gateways is that they have certain computing and storage capability, and can perform certain functions of edge computing.
- Routers: routers are nodes in the core network whose main function is to forward data packets to their destinations. According to the hardware configuration, certain computing functions can be added to routers. However, to be simple and without losing generality, in this paper, we call routers with computing functions as servers.
- Servers for cloud computing and service provisioning: servers for cloud computing have high networking, storage and computing resources. With the help of artificial intelligence and crowdsourcing techniques, cloud computing can process data efficiently and provides various of information and service. In addition, vehicle manufacturers, dealers, service providers (such as vehicle software providers, insurance providers), and service administrators (such as police station, traffic administration bureaus, etc.) may also have their own servers in the ITS system to provide legacy services (i.e., without security and privacy protection) and secure services (i.e., with the help of blockchain).

#### 3.1.2. Blockchain Overlay Network

The blockchain network is a virtual network consisting of entities in the ITS infrastructure and service provisioning layer. The use of overlay ensures the existing data communications and the legacy services will not be affected. Depending on the capabilities of nodes in the ITS infrastructure and service provisioning layer and their hierarchical topological organization, two types of blockchain nodes are introduced, which are organized in three types of interoperable chains. In addition, to keep the security and privacy of data, encryption and decryption of the data in the blocks are performed at the vehicular level blockchain.

##### Blockchain Nodes

We define two types of blockchain nodes. Depending on the capabilities and locations of the entities in the ITS architecture, gateways, RSUs, vehicles and servers can be set to one of these types and integrated with other specific functions.

One is full blockchain nodes. These nodes synchronize and maintain a full copy of the blocks and work as miners. They are responsible for updating data, broadcasting and verifying transactions in time. They provide computing power for blockchain network. Since these nodes can see all the transactions happened in the blockchain, only the authorized nodes with high capability can work as this type of nodes, such as RSUs, gateways, and servers in the core network.

The other is lightweight blockchain nodes. This type of nodes is similar to the simplified payment verified (SPV) node in Ethereum. The nodes do not need to store the data of the blockchain, they need only to keep a list of the block-headers. These nodes cannot validate transactions through mining, but they can find a transaction according to the Merkle tree and believe that the blockchain network has already validated the transaction. Since this type of blockchain nodes do not require high capabilities for computing and storing, any node in the ITS network can be set as this type of nodes.

Besides the basic blockchain functions, different functions are also added to different nodes:

- Gateway full blockchain nodes: some smart contracts can be deployed on this type of nodes. The nodes can visit and modify the smart contract pools. In addition, this type of nodes can also participate in both RSU-chain and gateway-chains, and is responsible for synchronizing data from RSU-chain to gateway-chain.
- RSU full blockchain nodes: some smart contracts can be deployed on this type of chains. For security consideration, all the vehicular nodes are not allowed to upload smart contract, they can only invoke the smart contracts deployed in the RSUs. RSU full blockchain nodes can participate in both RSU-chain and vehicle-chain, and are responsible for synchronizing data from vehicle-chain to RSU-chain.

Note that depending on the positions and needs of the ITS operators, the network entities, such as gateways, RSUs, routers and servers, etc. can be set as both full and lightweight blockchain nodes. Especially, vehicles can be set to both full and lightweight blockchain nodes, in order to be involved in the ITS deeply (i.e., involved in the blockchain network for more rewards) or just to use the secure services with privacy protection.

##### Hierarchical Organization of Blockchains

**Figure 2:**
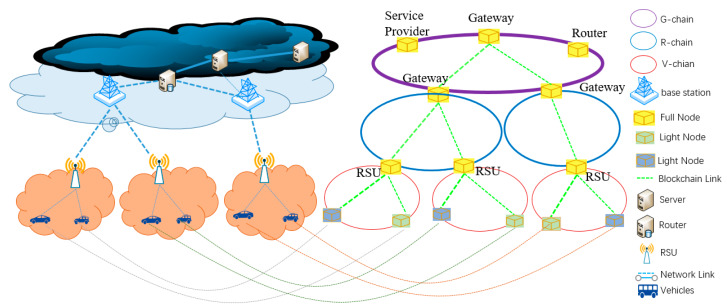
Hierarchical organization of blockchains in Ba-ITS.

A full blockchain node needs to store a copy of all the blocks since a genesis node has been created. For ITS, a large number of vehicles move all the time on roads and large amount of data will be involved in the ITS services. If only one blockchain is used, very high storage and computation capability will be required for each blockchain node. This might be not practical in ITS, especially for vehicular nodes. Therefore, we use multiple chains. Our purpose is to make vehicles concentrate on only the data in a certain area and take part in making consensus only in that data. Hence, considering the movement of vehicles and the architecture of ITS infrastructure, we designed a hierarchical blockchain structure with three layers of blockchains, namely vehicle-chain, RSU-chain and gateway-chain, as shown in Figure 2.

A vehicle-chain (V-chain) consists of the vehicular nodes communicating with the same RSU and the RSU itself, as shown in Figure 2. In order words, a RSU and the vehicles in its radio communicating area construct a V-chain. The RSU node acts as the genesis node and vehicles passing it may join the V-chain initiated by the RSU and involved in blockchain. Vehicles can obtain data and other services, as well as upload data through V-chain.

A RSU-chain (R-chain) consists of the RSU nodes connecting to the same gateway and the gateway itself, for example, the RSUs in the same base station coverage area and the base station itself or in a routing subnet of a gateway together with the gateway. R-chains can synchronize data from different V-chains involved by each RSU to the R-chain, which extends the visible area of data from each V-chain. The nodes acting as RSU and being able to participate in a R-chain node are those authorized by the ITS operator or its network operators, no private vehicles can join the R-chain, so a R-chain has higher privacy and security level than a V-chain, and smart contracts can be deployed in a RSU-chain for providing ITU services.

A gateway-chain (G-chain) consists of a gateway, router and server nodes. The purpose of the G-chain is to ensure the data exchange among different gateways, i.e., at the whole ITS level, so that tasks and rewards can be distributed to vehicles wherever they are, making it possible for mobile vehicles to use the ITS services provided by ITS. Similar to the R-chain, the nodes in the G-chain are those authorized by the ITS operator. Since the nodes in the G-chain are located in the core networks and have less security risks than RSU nodes, the G-chain has the highest priority in the blockchain network. Besides deploying smart contacts, G-chain can also access the smart contract pool, where smart contracts are maintained. The G-chain connects all the routers and servers in the networks, there is only one G-chain. In addition, as G-chain is at the highest level of the blockchain structure and maintains all the data, it acts as the backend sever exposing RPC to dApp, which can use e.g., the JSON RPC application programming interface (API) and the Web3 library to interact with the blockchain.

To realize the interoperability among the different levels of chains, we make some nodes join in two chains at different levels and synchronize data between them. For example, some RSU nodes can join in a V-chain and a R-chain simultaneously. When a block in the V-chain is generated, a smart contract will run automatically which calls the smart contract of data upload and puts the block in the R-chain. Similarly, the synchronization of data between R-chain and G-chain is realized by the gateway nodes joining simultaneously in the chains at these two levels. 

For all the above three levels of chains we use private chains instead of public chains. Since the public chains are fully distributed and may make the blockchain network very complex, lowering the efficiency and performance of the blockchain network. While private chains can process the transactions very fast, and the processing cost can be very low. In addition, private chains can provide better protection for privacy. In private chains, the writing and reading rights can be controlled, thus the data in the blockchain cannot be obtained by any node joining the chain and a node cannot upload data to blockchains arbitrarily. These make private chains better satisfy the requirements of ITS. 

Different from the traditional flat blockchain architecture, the hierarchical blockchain architecture allows chains at different levels to concentrate on different functions. The multiple V-chains allow nodes focus only the data in certain areas, which alleviate the burden of vehicle nodes and potentially improve the performance of the blockchain. The different access rights of nodes at different levels increase also the manageability of the blockchain, increasing the stability and feasibility of the proposed ITS system.

In addition, the hierarchical organization of the blockchains also enables the consensus on the data provided by the vehicles to be done locally by the RSU or vehicles configured as full blockchain nodes in the same V-chain. This can reduce the complexity of the consensus and the requirement on the computing power of RSU and vehicles whereas increase the speed of the consensus. The consensus on transactions happened at the G-chain and R-chain level is made by the full blockchain nodes at the corresponding level, which increases the scalability of the whole ITS system. Currently, we use the existing consensus protocols of blockchains to realize the consensus.

Furthermore, since the blockchain is introduced as an overlay, currently we assume that the sending and receiving transactions and messages caused by blockchain does not change greatly the data traffic model in the ITS. In other words, we depend on the current network planning and traffic optimization mechanisms to deploy and optimize the numbers and locations of RSUs, Gateways and Routers etc. in the ITS network infrastructure layer and the cloud computing and service provisioning layer. Traffic modelling in Ba-ITS is our future work.

##### Encryption of Data in the V-Chains

In the traditional blockchain, although the identities of users are anonymous, the data in the blocks are transparent for all the users in the blockchain, and the traceability of data is possible. In our system, to control the visibility of different data and better protect the privacy of users, we encrypt the data stored in V-chain. Since the nodes in R-chain and G-chain are all authorized by the operators, and the data on these chains will not directly visited by vehicular nodes, the data on the R-chain and G-chain will not be encrypted. 

The RSUs’ public and private keys are used for the encryption and decryption. Considering the fast movement of vehicles and the upload or query of data may be not very often, a vehicle will first request the RSU’s public key from the local RSU before uploading data to a V-chain through transactions (see Section 3.2.3), then encrypt the data. Then the encrypted data will be put in V-chain. In practice, the RSUs’ public keys can also be broadcast through the route advertisements, and each vehicle can obtain the public key of a RSU when it enters the radio communication area of the RSU. 

Through encryption, the access of data can only be through RSUs. No vehicles can access the data randomly. During the synchronization, the data is decrypted by the RSU and put in the corresponding R-chain. 

### 3.2. Service Provisioning Based on Smart Contract

To be able to share data among vehicles and ITS and meanwhile protect the users’ privacy as much as possible, the data exchange and other services are based on transactions and smart contract. In Ba- ITS, vehicle may participate in establishing and extending different V-chains as they pass different RSUs, at the same time obtaining services and providing data for other vehicles in the network. An incentive mechanism is introduced in Ba-ITS in order to encourage vehicles to join the blockchains.

#### 3.2.1. Smart Contracts

One of the most important advantages brought by blockchain is smart contracts, which are programs executing automatically in blockchain driven by events—when all the prescribed terms are fulfilled, certain actions will be executed. In essence, smart contracts provide a means to program and operate the data in blockchains. In our system, we use smart contracts to provide services by controlling and exchanging information and resources among users and service providers, such as data query, data provisioning, software updating, insurance services and traffic rule violation fine etc., since the logic of the services in ITS can be described with certain conditions and potentially involve different entities. In addition, the use of smart contracts can reduce the overhead of time and monetary cost, and also eliminate the mistakes and misbehaviours of human beings, increasing the trust of services. Moreover, smart contracts make it fast and easy to deploy new services. Deploying new services might simply mean adding new contracts in the contract pools. The conditions for executing smart contracts can be decisions from other algorithms, for example machine learning, or proximity-based (physical, social or temporal) context computing, or consensus-based comparison and selection etc. Especially, by binding contracts, complicated services can be provided.

Generally, two types of smart contracts are used in our architecture: (i) data query and uploading related smart contracts, and (ii) ITS service related smart contracts. The former is deployed in R-chain, since RSUs are deeply involved. Deploying on R-chain can reduce the service response time. The latter is deployed in G-chain, since all the information related to the services is maintained by the service providers’ servers and generally this type of services do not have strict time requirement.

However, for security reasons, the smart contract pool is maintained at the G-chain. Namely only entities in the G-chain (e.g., a service provider) can add and obsolete smart contracts by accessing or adding an item in the pool after being granted a license from the ITS operator and acting as the manager of the corresponding smart contracts. In the following we will describe the incentive mechanism and two examples of service providing procedure, where smart contracts are used.

#### 3.2.2. Incentive Mechanism

In addition to the inherent incentive mechanisms of blockchain, we use an incentive mechanism based on reward in Ba-ITS to encourage vehicles to join the blockchain. Vehicles will pay a certain amount of units when using the services from Ba-ITS while get a certain amount of units as reward when providing data or other services to Ba-ITS. The incentive mechanism is realized by using smart contracts which may be deployed on RSUs or gateways depending on services. The price of a service will be dynamically adjusted according to the number of times the service has been used and the system cost, controlled by the corresponding smart contract.

Suppose *F_1_* is the fee charged for querying data, *F_2_* is the fee rewarded for uploading data, and *F_3_* is the intermediary fee charged by RSU or gateway running a smart contract. The smart contract will adjust the fees periodically (e.g., at every night) according to the profit and loss of RSUs or gateways, to ensure that *F_3_* = (*F_1_*− *F_2_*) × α%, is greater than the power and device depreciation cost of RSUs or gateways. Depending on the consumption of power and device depreciation in the certain period (e.g., daily), the coefficient α can be adjusted. Finally, the fee *F_1_* and *F_2_* for the next period (e.g., the next day or month) will be adjusted according to the size of the reward pool of the corresponding RSU or gateway nodes. 

#### 3.2.3. Example Services

In order to show the feasibility of the proposed Ba-ITS, we implemented two types of services typical for ITS, data query and upload, and route planning. The former provides a basic service for other services and may have certain response time requirement, the latter involves the gateway level chain (G-chain) and provides a new way of providing the same services as in the legacy ITS. 

##### Information Query and Upload

Information query and upload will be one of the basic services provided by the future ITS. The information can be used by vehicles to assist driving, or by the system or a 3rd party to develop and provide new services. For example, vehicles may need certain data from a specified area. They can request them from the ITS system and obtain the data after paying (e.g., through an automatic bill). Vehicles on the road can collect data by utilizing their sensors and upload it to the system, gaining some reward which can be used later to request data or other services. 

**Figure 3:**
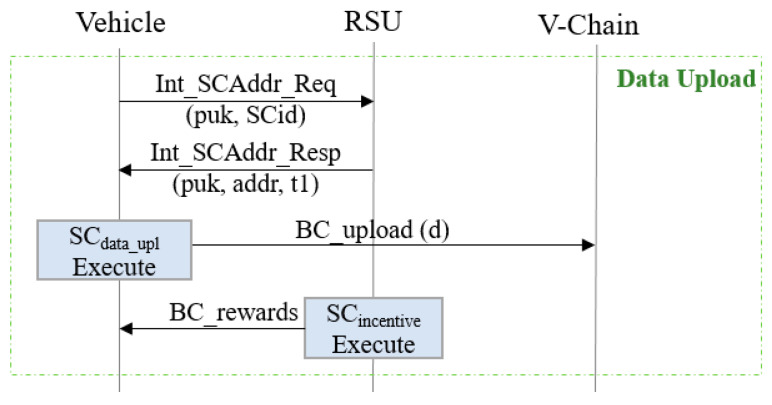
Vehicles upload data to the V-chain.

In Ba-ITS, the data upload and query are realized by using smart contracts. As shown in Figure 3, when a vehicle wants to upload data, e.g., about road and environment conditions or vehicle states, it will first ask the address of the corresponding smart contract (denoted as SC_data-upl_), then invoke the smart contract, which uploads data to the V-chain. Then the data will be synchronized to R-chain and G-chain (see also Section 3.2.3). When the data is successfully uploaded, the incentive mechanism will be triggered, which puts certain credits to the vehicle’s account. To be clear, in our paper we use messages begin with Int_ to denote the information exchanges using legacy network protocols (i.e., Internet protocols); messages begin with BC_ to denote the transactions and messages of blockchain, such as those triggered by the execution of a smart contract.

**Figure 4:**
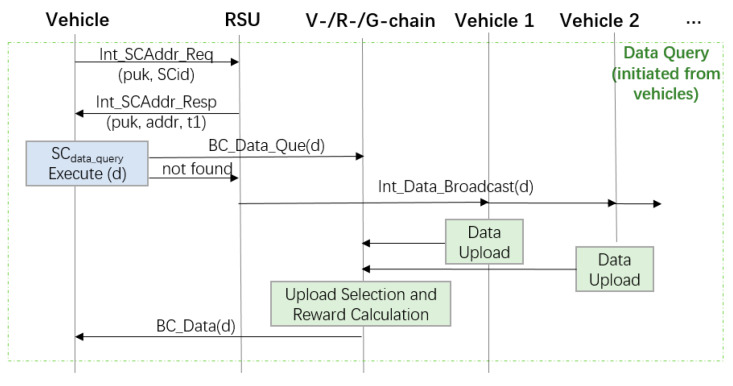
Data query initiated by a vehicle.

Figure 4 illustrates an example of data query initiated from a vehicle. When a vehicle needs certain data during driving, it will first ask the nearby RSU the address of the smart contract for data query (denoted as SC_data-query_). When the address is obtained, the vehicle will invoke the smart contract, which looks up the data in the blockchain. If the requested data already exist in the blockchain, the data will be sent to the vehicle through a transaction. Otherwise, a procedure of looking for the data will be triggered, in which the RSU broadcasts the data requirement in its radio coverage area. 

As a result, one or several vehicles might be willing to upload the requested data. In our currently system, the data uploaded from the first vehicle will be selected. However, a scoring mechanism can be used to select the vehicle with highest reputation record. If no vehicle is willing to upload data, a message with no data will be returned to the vehicle requesting data. 

##### Route Planning

In many cases, information about a large area (compared with the coverage of a base station) is needed in order to provide services such as suggesting an optimum driving path. Therefore, information exchange at the G-chain level is essential. This example shows how the proposed system can provide services conveniently to users by using dApp. In this example, a user uses a dApp interface to provide the starting point and destination, the system will send the optimum route according to a certain strategy (e.g., the shortest time or distance). According to the changes of the road conditions, new results can be sent to the user.

**Figure 5:**
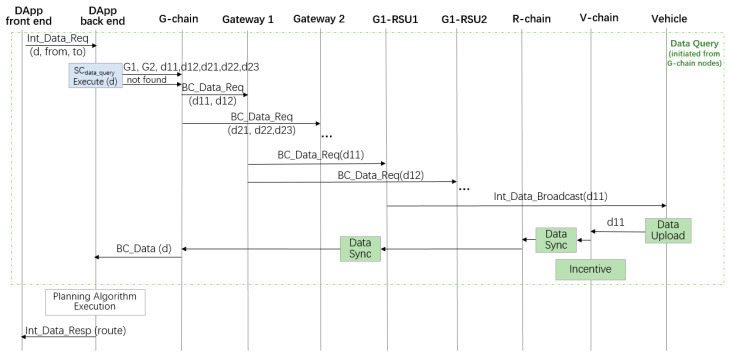
Data query when no data are found in the blockchain.

The route planning service includes the procedure of data query initiated from a node in the G-chain. As illustrated in Figure 5, a user uses the dApp client to send his route request, describing the starting point and destination. After receiving a route plan request, the dApp server will request the needed data at certain locations for calculating the optimum route from the G-chain (e.g., data d11and d12 from Gateway 1 and d21, d22, d23 from Gateway 2 are needed in Figure 5). If not all the requested data can be found, the G-chain will initiate messages requesting the missed data (e.g., from Gateway 1 and Gateway 2). Correspondingly, Gateway 1 and Gateway 2 will request the data from the RSUs in their coverage area. Then the RSUs will broadcast the required data. Vehicles who have or will collect the data will initiate the data upload procedure as discussed before and get some reward. When all the request data are stored in the G-chain, the smart contract will send the data to the dApp server. The dApp server will run the route planning algorithm, and the result will be sent to and displayed in the user’s dApp interface. Note that in Figure 5, we omitted the messages send from Gateway 2 and G1-RSU2 (i.e., RSU2 of Gateway 1). They execute the same actions as Gateway 1 and G1-RSU1.

Most of the current navigating applications require the location information of vehicles and the third party service provider can collect users’ activity and itinerary information without notifying users. For example, in the current GPS-based route planning application, a central server first requires a vehicle’s starting point and destination, then calculates an optimum route for the vehicle by using the snapshots of different paths collected by the GPS. During this procedure, the user’s information, including users starting point, destination and real-time location, travelling paths and time etc., will be backed up in a server. This exposes users’ privacy greatly, whereas in the route-planning service based on Ba-ITS, GPS can still be used for positioning. However, in Ba-ITS the calculation of route is performed by a smart contract. No servers of the service provider or a third party are involved for storing and processing a user’s data. Together with the anonymity of blockchain, no private information of users can be identified.

Ba-ITS changes the way of data acquisition and storage. By using the proposed architecture, users will not have the misgivings of exposing their privacy or misusing the data they provide. Together with the incentive mechanisms, uses will actively make contributions to the systems.

### 3.3. Features of Ba-ITS

The Ba-ITS can provide the following features to users with the help of the inherent anonymity, distributed trust, and data integrity of blockchain, and at the same time stimulate users to be involved in the data services of ITS.

#### 3.3.1. Flexible Service Provisioning

The use of smart contracts in Ba-ITS provides a flexible way for introducing new services. The smart contract pool and the corresponding addresses of each smart contract provide an interface for service providers to program and operate the data in the blockchain and the system to deploy new services quickly and efficiently. 

#### 3.3.2. Scalable System

The hierarchical organization of chains enables the scalability of the system. The synchronization mechanism between chains realizes a kind of interoperability among chains and enables the visibility of data in the whole ITS system.

#### 3.3.3. Privacy Protection

Since only vehicles’ blockchain addresses (e.g., public keys) are involved in the data exchanges, users’ personal information is separated from the data provided by users. In addition, the servers from the 3rd parties (e.g., service providers) cannot change the data stored in the Ba-ITS system. This reduced greatly the risks of misusing users’ data. Furthermore, since the blockchain is introduced as an overlay network in Ba-ITS, the compatibility to legacy systems and vehicular networks are kept. This provides a user-specified privacy protection. Vehicle users can specify if to use blockchain or what data are private. 

#### 3.3.4. Trusty System Administration

The blockchain technology enables the trace of data exchange and usage in the network. This makes it possible to trace the network activities in the event that privacy leaking happens. In addition, since the fulfillment of the smart contracts is verified by the trustworthy nodes and execute automatically, the mistakes and misbehaviours of human beings can be eliminated. Thus, the usage and administrative of services are trustworthy in Ba-ITS. 

#### 3.3.5. Security

The blockchain technology has integrated strong security techniques, such as distributed ledger and validation, proof of work and encryption, etc. This ensures the data provided by users cannot be tampered with in the networks, especially by the service providers which can be a big security problem in the legacy systems. Moreover, the encryption mechanism used in the V-chain provides also an access control mechanism. The data uploaded by a vehicle cannot be obtained by any nearby vehicle. Moreover, different types of blockchain nodes can be supported in Ba-ITS. When different access rights are given to the different types of nodes, a potential access control mechanism can be realized.

Besides the technical features, using Ba-ITS may bring big economical benefits to users, ITS operators and the society. The owners of vehicles can obtain rewards or more services by contributing the extra on-board computing and memory etc. resources of the vehicles. This may eventually save the energies of our planet. The inherent feature of autonomous running of blockchain networks can greatly reduce the operation and maintenance of the ITS system for the operators. In addition, the ITS operators may also obtain financial rewards by issuing tokens and cooperating with other industries or service providers. 

However, as mentioned in Section 3.2.3, currently we have not used a mechanism to deal with the erroneous information provided by vehicles in Ba-ITS. We are now working at a scoring mechanism, in which the vehicle with highest reputation record of providing data will be selected to put the data in the blockchain and get more reward.

## 4. Implementation and Test Results 

We implemented a prototype of Ba-ITS based on Ethereum and measured the time of data query and upload. To compare the performance with that in the current ITS infrastructure, we simulated a vehicular network in ns-3 with Open Street Map (OSM) [32] and Simulation of Urban Mobility (SUMO) [33]. In the following, we will first describe the implementation of the prototype, then elaborate the test results. 

### 4.1. Implementation of Ba-ITS

Ethereum, as a secure decentralized and generalized transaction leger enables programmability to the blockchain. The introduction of smart contract makes it possible and easy for blockchain technology to be used in different areas. Therefore, we implemented private chains based on Ethereum and established a prototype of Ba-ITS, including the incentive mechanism and the data query and upload etc. services. We set up three hierarchical chains and generated accounts using Ganache [34] for vehicles, RSUs and gateways. We developed a dApp by using the Truffle [34] framework to connect drivers with blockchains. We implemented smart contracts using Solidity [23], and deployed them on RSUs and gateways. We used the JSON RPC application programming interface (API) and the Web3 library to implement the interaction with the blockchain.

**Figure 6:**
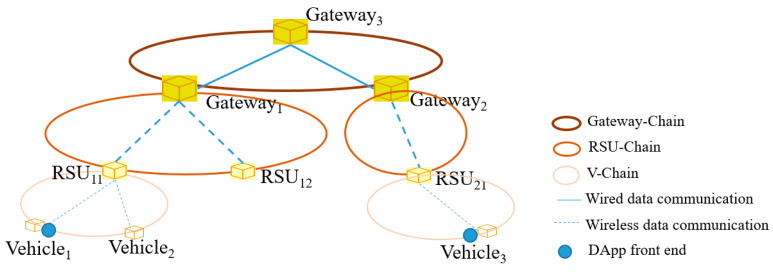
Topology of a vehicular network with ten blockchain nodes.

Figure 6 shows the topology of the network we established. Nine blockchain nodes constructed five blockchains at three levels. We simulated the V-chain, R-chain and G-chain using Ubuntu 18.04, Windows 7 and MacOS, respectively. In addition, two geths (go-ethereum) clients were generated on RSU_11_, RSU_12_ and RSU_21_ joining the V-chain and R-chain respectively. Similarly, two geths are generated on Gateway_1_ and Gateway_2_ too, joining R-chain and G-chain respectively. In addition, a dApp runs on Vehicle_1_.

**Figure 7:**
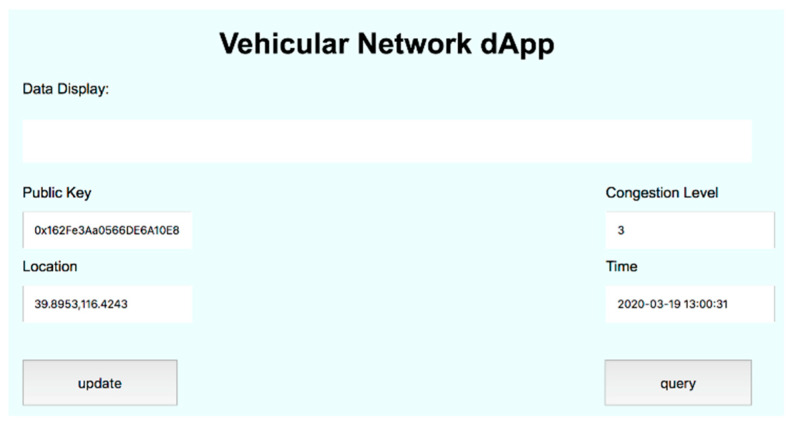
Vehicles can use dApp to query and upload data.

In order to test the basic services, we implemented a dApp with user interface for vehicles to query and upload data, input parameters and illustrate the results of the route planning service. Figure 7 illustrates the user interface running on Vehicle_1_, where the location, public key, and the obtained or upload data etc. can be displayed.

**Figure 8:**
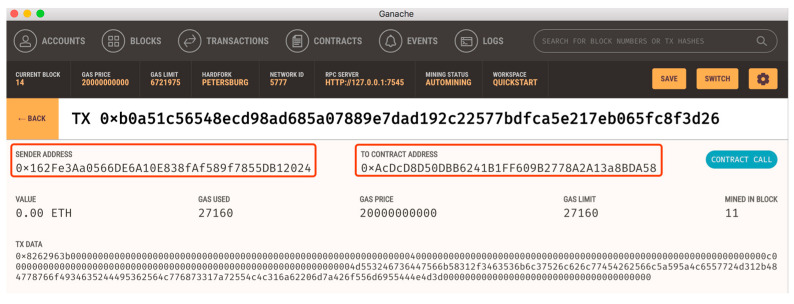
Data in the V-chain blocks are encrypted.

To test the accessibility of the data stored in the blockchain, we start Vehicle_2_ as a full blockchain node which joins the mining and stores blocks. We check the blocks through Ganache. As shown in Figure 8, the data in the blocks has been encrypted and cannot be read directly, although the blocks are stored locally in the vehicle. 

### 4.2. Data Response Time

Here data response time means the elapsed time when a vehicle begins to query data until it obtains the data from the ITS. First we test the time required for a vehicle to query data that already exist in the blockchain. We let Vehicle_1_ upload some test data to the network in advance, then measure the time when Vehicle_3_ starts to query the data and when it obtains the requested data, and calculate the time difference. 

**Figure 9:**
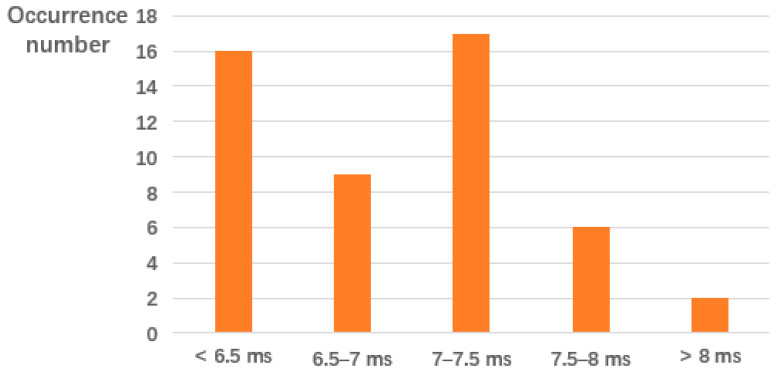
Distribution of data response time when the requested data exist in Ba-ITS.

Figure 9 illustrates the distribution of the time for 50 times of independent tests. Here we can see that the data query time is less or equal than 7.5ms for most cases (i.e., 84%). 

**Figure 10:**
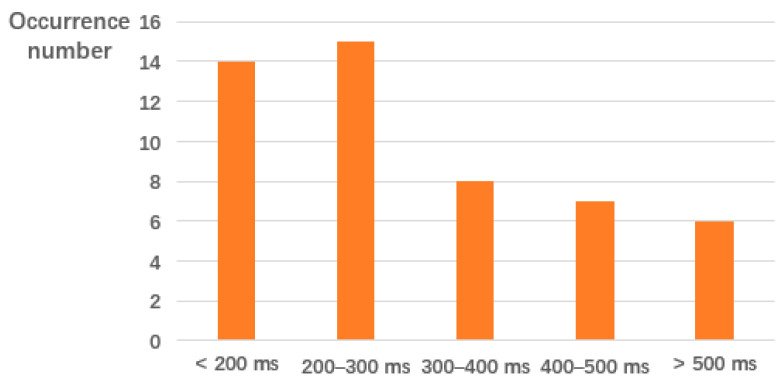
Distribution of data response time when the requested data do not exist in Ba-ITS.

In the second test, we measure the time when the requested data have not existed in the network. In this test, we let Vehicle_3_ to request the data. Then Vehicle_2_ will upload the data. Figure 10 shows the result of tests for 50 times. Here the “difficult parameter” in Ethereum is set to 0x10000. We can see the data response time is much longer than that when the request data is already on the chain. To analyze the reason, we measure the time for data onto chain, as discussed in Section 4.3.

### 4.3. Time of Data Addition to the Chain

Since the data query time is closely related to the data upload time, we first analyze and measure the data upload time. As mentioned in Section 3.2.3, the time for uploading data and putting in the blockchain (so that the data can be accessed by others) consists of four parts, namely the time for: (i) information exchange between a vehicle and a RSU; (ii) invoking a smart contract, which causes a transaction in a V-chain; (iii) data synchronization between V-chain and R-chain, which causes a transaction in a R-chain; (iv) data synchronization between R-chain and G-chain, which causes a transaction in the G-chain. If we neglect the time for processing messages, the time for part (i) is the time used for two network message exchanges, in our case, it will be the data transmission time of two wireless hops. Part (ii) consists of two components, the first is the time for one-way message exchange (i.e., RPC, remote procedure call which is approximate to one wireless hop); the second is the time for a transaction exchange, then being added to a block and mined until attached to a blockchain. To be convenient, we call the second part the time for data onto chain. Parts (iii) and (iv) are the same as the second component in part (ii), namely the time is used for adding data to the chain. Hence, the time for data addition to the chain decides mainly the time needed to upload for data, and we measured the time for data addition to the chain with the help of the Ethereum developing tools. 

Table 2 illustrates how the time for data addition to the chain varies with different “difficulty parameters” set for controlling the speed of mining blocks in Ethereum (i.e., how difficult it is to find a hash below a given target). Here we measured the time in machines with two capabilities, one is with CPU of 2.6 GHz Intel Core i7, 6 cores, 16 G RAM, and macOS (denoted as CPU1 in Table 1), and the other is with CPU of 2.5 GHz Intel Core i5, 4 cores, 6 G RAM and Windows 7 (denoted as CPU2). 

Here we can see that the speed of mining blocks depends on the hardware of nodes and the “difficult parameters” used in Ethereum. The time for adding data to the chain depends closely on the mining speed. In practice, since the nodes in the network, such as RSUs are dedicated for the data exchange, we can set difficult parameters for different applications, such the response time that can be controlled. 

### 4.4. Comparasions with Traditional Vhicular Networks

In order to estimate the overhead brought by the blockchain, we implemented a vehicle network in the simulation environment ns-3 to test the time of data transmission and execution of route planning service in the traditional vehicular networks. To make the vehicular network and the route planning service as realistic as possible, we use OSM and SUMO to generate vehicular nodes for ns-3. OSM contains road information that is generated and validated by satellite images and GPS traces, and is commonly regarded as the highest quality road data publicly available today. SUMO is an open-source traffic simulator with continuous space and discrete time. SUMO is capable of importing maps in multiple formats, including OSM. In our simulation, we use OSM to create the real-world map, and use SUMO to generate vehicle nodes and traffic, and mobility model in the real-world road maps. The results were exported to ns-3, where the LTE and IPv4 modules are used. Table 3 shows the parameters of ns-3 used in our simulations. 

**Figure 11:**
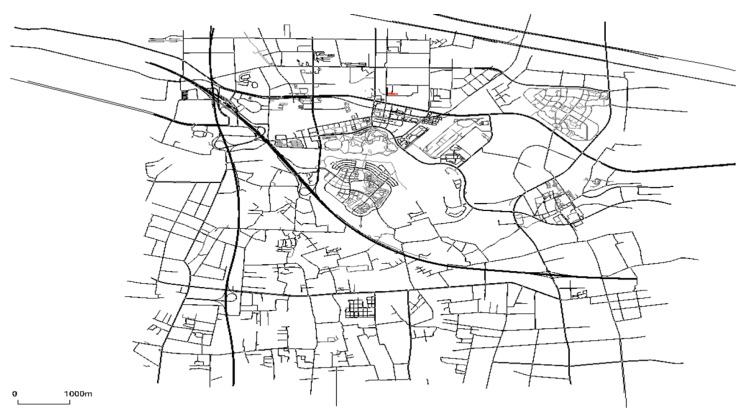
Map used in ns3 for route planning.

In the simulation, we select the map of Huangpu District of Shanghai City with an area of about 9.3 km × 8.0 km, transform it through SUMO, and generate the vehicle’s motion track through SUMO’s own software, as shown in Figure 11.

We implemented the A* algorithm [35] in our simulation environment (i.e., in a “route planning server node) for calculating the route. During the simulation, a source and destination on the map was randomly selected. In addition, the same network topology as Ba-ITS is used, namely, the route planning request is sent to the server via vehicle- > RSU- >base station- > server, and the route planning result is sent from the server to the vehicle via in the reverse way.

**Figure 12:**
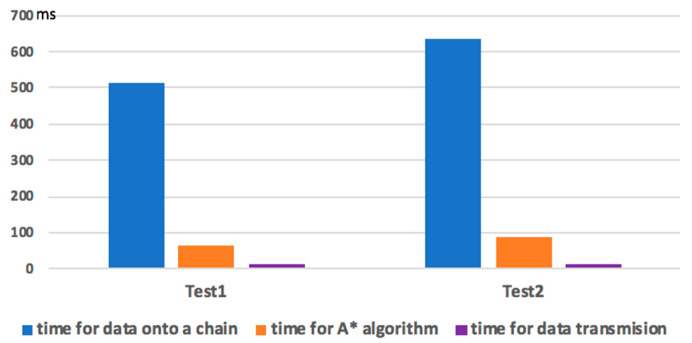
Comparisons of service response time (milliseconds).

We measured the service response time, i.e., the time between the route planning request with parameters of starting point and destination are sent to the network and when the planned route is sent back to the vehicle. To compare the service time in the legacy vehicular network and in Ba-ITS, we measured the time used in different procedures. Figure 12 illustrates the results for two tests. Here the “difficulty parameter” of Ethereum is set to 0x10000.

Here we can see that the majority of time are spent adding data to the chain. However, the service time for route planning (i.e., time for adding data to the chain plus the time for A* algorithm execution) will be less than 1 second, which can satisfy the needs of the users. In practice, the capability of nodes in the blockchain can be higher, so the service response time can be improved. 

In addition, depending on the services, network nodes can also control the speed of data addition to the chain for different types of data, and make efficient use of computation resources. Currently, the capability of computing power has developed greatly and different levels of computation efficiency (e.g., considering cost, speed and power consumption etc.) can be achieved in practice by using ASIC, FPGA, GPU [36], etc.

## 5. Conclusions and Future Work

In this paper, we propose an architecture for ITS with a blockchain overlay network. The proposed architecture uses blockchain as a trust infrastructure to protect users’ privacy and provide trustworthy data and services. Based on the architecture, blockchain’s advantages such as distributed trust, anonymity, data integrity are used in providing services in ITS. Thus, services provided by the network cannot damage users, and users will not expose their privacy when providing data or using services from the ITS system. In addition, malicious behaviours of both users and service providers can be traced.

We have designed a hierarchical blockchain for the proposed Ba-ITS, which guarantees scalability in Ba-ITS and services. Interoperability among different levels of blockchains has been elaborated. In addition, new services can be deployed based on smart contracts in the proposed Ba-ITS, which provides a flexible, fast and trustworthy way for service provisioning. In addition, an incentive mechanism according with blockchain’s nature has been designed in the proposed Ba-ITS, to encourage users and vehicles to be involved in the ITS, contributing data to the ITS and obtaining secure and trustworthy services from the ITS.

As future work we plan to test the proposed Ba-ITS on a larger scale. We will evaluate the performance of the proposed system thoroughly, for example, using more and different types of blockchain nodes, evaluating the relationship between service response time and the network scale. Optimizing and refining the proposed architecture is also our future work, such as setting criteria for selecting RSUs or gateways that can join two chains, etc.

## Figures and Tables

**Table 1:** Comparisons of the current work on secure ITS.

	Traditional ITS Infrastructure			Blockchain-Based ITS		
	[10]	[11]	[13]	[5]	[26]	[27]
Privacy	User can be traced	User can be traced	User can be traced	Anonymity	Anonymity	NA
Trust	Signature	Signature	NA	Yes	Yes	Yes
Authentication	Yes	Yes	Yes	Yes	Yes	Yes
Accountability	Yes	Yes	Yes	Yes	Yes	Yes
Attacks Protection	Bogus message attacks	Tampering	Spoofing	NA	NA	NA
Data Consistency	NA	NA	NA	Yes	Yes	Yes
Confidentiality	NA	Yes	Yes	Yes	Yes	No
Integrity	Yes	Yes	Yes	Yes	Yes	Yes
Non-repudiation	Yes	Yes	NA	Yes	Yes	Yes
Access Control	Yes	Yes	No	NA	NA	NA
Scalability	NA	NA	NA	NA	NA	NA
Implementation Limitations	Require to collect the behavior of vehicles	Require to register in a group	Broadcasting delay of messages	High computing power and memory requirement	High memory requirement	High memory requirement

**Table 2:** Time for data onto chain.

Difficulty Parameter	Time (ms/block)	
	CPU1	CPU2
0x5FFFFA	12000	15000
0x99999	1075.28	1258.07
0x40000	433.34	522.53
0x30000	292.30	325.59
0x20000	287.99	324.27
0x10000	242.95	256.53

**Table 3:** Parameters setting for ns-3.

Parameters	Values
Simulator	ns-3(version 3.29)
Simulation time	900 s
Simulation area	9320 m × 8099 m
WLAN Protocol	802.11 g
Data rate	1 Mbps
Mobility model	Constant Position Mobility Model
Signal propagation loss model	Range Propagation Loss Model
Delay model	Constant speed propagation delay model
Radio transmission range	300 m
Radio frequency	2.4 GHz
Vehicle speed	40–50 km/h
